# Case Report: End-Stage Recurrent Glioblastoma Treated With a New Noninvasive Non-Contact Oncomagnetic Device

**DOI:** 10.3389/fonc.2021.708017

**Published:** 2021-07-22

**Authors:** David S. Baskin, Martyn A. Sharpe, Lisa Nguyen, Santosh A. Helekar

**Affiliations:** ^1^Kenneth R. Peak Brain and Pituitary Tumor Treatment Center, Department of Neurosurgery, Houston Methodist Neurological Institute, Houston, TX, United States; ^2^Department of Neurosurgery, Houston Methodist Research Institute, Houston, TX, United States; ^3^Department of Neurosurgery, Weill Cornell Medical College, New York, NY, United States

**Keywords:** magnetic resonance imaging, contrast enhanced tumor, compassionate use treatment, radiation-type tumor necrosis 2, oscillating magnetic fields

## Abstract

Alternating electric field therapy has been approved for glioblastoma (GBM). We have preclinical evidence for anticancer effects in GBM cell cultures and mouse xenografts with an oscillating magnetic field (OMF) generating device. Here we report OMF treatment of end-stage recurrent glioblastoma in a 53-year-old man who had undergone radical surgical excision and chemoradiotherapy, and experimental gene therapy for a left frontal tumor. He experienced tumor recurrence and progressive enlargement with leptomeningeal involvement. OMF for 5 weeks was well tolerated, with 31% reduction of contrast-enhanced tumor volume and reduction in abnormal T2-weighted Fluid-Attenuated Inversion Recovery volume. Tumor shrinkage appeared to correlate with treatment dose. These findings suggest a powerful new noninvasive therapy for glioblastoma.

## Introduction

For glioblastoma (GBM), the most common malignant tumor of the brain in adults, treatment outcome remains dismal. In over 40 years median survival has only shown modest improvement (1), and standard of care treatment often has negative impact on quality of life (2). Treatment including radiation and chemotherapy takes a heavy toll. Frequently patients cannot tolerate the completion of the prescribed chemotherapy cycles. Thus, there is a great unmet need for a completely different therapeutic approach with better outcome and less toxicity.

A new FDA-approved treatment involving electric fields alternating at 200 kHz called Optune™ therapy is now available for recurrent GBM as monotherapy and in combination with temozolomide for newly diagnosed GBM (3, 4). It is also being tested in clinical trials for other cancers. Its hypothesized mechanism of action involves disruption of tubulin dimers, mitotic spindles, and cell division by electric field-induced dipole alignment and dielectrophoresis (5). It has a modest effect on survival, increasing median overall survival by 0.6 month in recurrent GBM (3), and in newly diagnosed GBM by 31% (4). Even this modest effect is encouraging for patients.

It has been shown that electromagnetic fields (EMF) produce anticancer effects *in vitro* (6, 7). We have conducted preclinical experiments with a new noninvasive wearable device known as an Oncomagnetic device that generates oscillating magnetic fields (OMF) by rotating strong permanent magnets (8, 9). The OMF generating components (oncoscillators) of the device can be attached to a helmet and treatment with the device does not require shaving the head. Using the oncoscillators of the device and specially devised patterns of magnet rotations we have produced strong selective anticancer effects in patient derived GBM and xenografted mouse models without causing adverse effects on cultured normal cells and normal mice (10–12). The mechanism of action of OMF differs from Optune™ and involves disruption of the electron transport in the mitochondrial respiratory chain causing elevation of reactive oxygen species and caspase-dependent cancer cell death (10–12).

Here we report evidence of treatment response in the first patient to ever receive this therapy with an untreatable left frontal GBM, treated with a wearable Oncomagnetic device in an FDA-approved Expanded Access Program.

## Methods

### Case Description

The patient is a 53-year-old man who first presented with altered mental status in May 2018. Imaging studies documented a large tumor in the left frontal lobe extending across the midline into the right frontal lobe, with diffuse and extensive infiltration through the corpus callosum. There was mass effect and severe edema. He was taken to the operating room on June 4, 2018, where he underwent left frontal craniotomy and radical excision of the tumor. The tumor was histopathologically confirmed as GBM. At the time of the surgery, the excision extended across the midline into the right frontal lobe. He was enrolled in a herpes simplex virus-thymidine kinase gene therapy program and received viral injection during surgery per protocol. In addition, per protocol, and as standard of care, he received concomitant radiation therapy and chemotherapy with temozolomide.

In August 2019, the patient presented with an area of contrast enhancement on MRI scan along the left ventricle. At first this was thought to be a treatment effect. This area progressively enlarged. Evaluations done before OMF treatment initiation on January 16, March 3, and April 15, 2020, demonstrated a clear recurrence. The tumor abutted the ventricle and there was evidence of leptomeningeal spread. The patient had already had radiation therapy and chemotherapy and the tumor was now progressing. The presence of leptomeningeal disease portends poor outcome, with median survival of 3.5 to 3.9 months (13).

Because of inadequacy of any standard of care options he was enrolled in an FDA-approved Expanded Access Program (EAP) for compassionate use treatment with the Oncomagnetic device. He signed an informed consent on April 15, 2020. The EAP study was carried out under a protocol approved by the Houston Methodist Research Institute Institutional Review Board.

### Oncomagnetic Device

**Figure 1:**
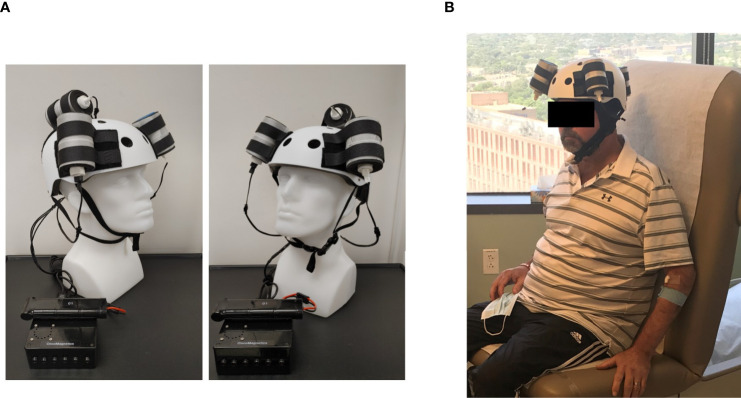
Oncomagnetic Device. **(A)** Device helmet with 3 oncoscillators securely attached to it. The oncoscillators are connected to a controller box powered by a rechargeable battery. **(B)** The patient wearing the device helmet with three oncoscillators attached.

The Oncomagnetic device consists of 3 oncoscillators securely attached to an acrylonitrile butadiene styrene helmet and connected to a microprocessor-based electronic controller operated by a rechargeable battery (**Figure 1**). Further details regarding the device are given in the **Supplementary Appendix**. Based on a finite element model-based calculation of the spread of the field and the size and magnetization of the rotated diametrically magnetized neodymium magnets, we estimated that the combined effective field (at least 1 mT in strength) of the 3 oncoscillators covered the entire brain, including the upper part of the brain stem.

### Oscillating Magnetic Field Treatment

The treatment consists of intermittent application of an OMF that needs to be generated by rotating permanent magnets in a specific frequency profile and timing pattern to be effective. The patient received this treatment initially in the Peak Center clinic under the supervision of the treating physician and the Principal Investigator (DSB) of this study for the first 3 days. The dose was escalated over this period as follows. On the first day, the treatment was for 2 hours with a 5-min break between the first and the second hour. On the second and third days, it was increased to 2 and 3 2-hour sessions, respectively, with 1-hour breaks between the sessions. The patient’s spouse was trained in the use and care of the device on these days. After this initial supervised phase, the treatment was continued at home unsupervised with the same regimen as on the third day, above. The spouse was instructed to maintain a daily log of the conduct and progress of treatment, and any observed treatment and adverse effects.

### Clinical Evaluations and Neuroimaging

The patient was evaluated clinically by the treating physician on each of the 3 days that he received treatment in the clinic and 7, 16, 30 and 44 days after initiation of treatment. Magnetic Resonance Imaging (MRI) scans were done on Days 1, 3, 7, 16, 30 and 44. The Day 1 scan was done before initiation of treatment. All other scans were done after treatment initiation. The treatment was paused on Day 37 because of an unfortunate but unrelated severe closed head injury (CHI). MRI scans were done on a Siemens Magnetom Terra 7T scanner. MRI scans included T1 magnetization prepared rapid gradient echo scans with and without gadolinium contrast, and T2-weighted Fluid-Attenuated Inversion Recovery (FLAIR), T2-weighted Turbo Spin Echo, Diffusion Weighted Imaging, Susceptibility Weighted Imaging, proton Magnetic Resonance spectroscopy and Diffusion Tensor Imaging scans. Treatment effect on contrast-enhanced tumor (CET) was evaluated according to the response assessment in neuro-oncology (RANO) criteria for clinical trials (14). In addition, an automated software-based method developed in house was used to objectively calculate the CET volume (see below and **Supplementary Appendix**).

### Data Analysis

Post-contrast T1 anatomical and T2-FLAIR MRI scans at each of the 6 time points were used to determine changes in contrast-enhanced tumor (CET) volume and non-enhanced tumor infiltration, respectively, before and after initiation of treatment. Information on image processing, data normalization and plotting are given in the **Supplementary Appendix**. Values obtained from pre-treatment clinical scans taken at 2 time points over 3 months before enrollment of the patient were also plotted on the same graph. Because this is a single patient case report, we could not perform any meaningful statistical analysis. However, to obtain a semi-quantitative assessment of the significance of the trend seen with treatment, we analyzed the changes in CET volume using Bayesian logic, given the observed increasing trend at two pre-treatment time points. Accordingly, we assumed that the chance of increase, decrease and no change in the rate of tumor growth was the same at each time point after treatment initiation to calculate the probability of a decrease at each post-treatment initiation time point.

## Results

The patient received OMF treatment with the Oncomagnetic device for 36 days. The treatment regimen was changed at various times during this period based on the caregiver reports and clinical findings, as described below.

### Clinical Findings

After the initial 3 days of supervised treatment, the patient was seen again by the treating physician in the outpatient clinic on Day 7 from the start of treatment. Because of inattention at baseline, the patient was having difficulty with the length of treatment sessions. They were reduced to 2 hours/day Monday through Friday with Saturday and Sunday off. The Day 16 clinical examination revealed that he was tolerating the treatment sessions well, so they were increased to a total of 3 hours/day (in one-hour increments with 5 min breaks) Monday through Friday and the weekends off. On Day 30 visit, the patient reported headaches related to transient hypertension for which he was taking medication. The treating physician increased blood pressure medication (Valsartan) with improvement. The treatment was paused on Day 36 because of a closed head injury from a fall. Whether the fall was related to the treatment in any way is uncertain. It is worth noting, however, that the patient had experienced several falls before initiation of treatment. At the last follow-up on Day 44 the patient was admitted to the inpatient unit for evaluation of closed head injury and underwent detailed assessment. There were no serious adverse events reported during treatment. The patient’s caregivers reported subjective improvement in speech and cognitive function.

### MRI Findings

**Figure 2:**
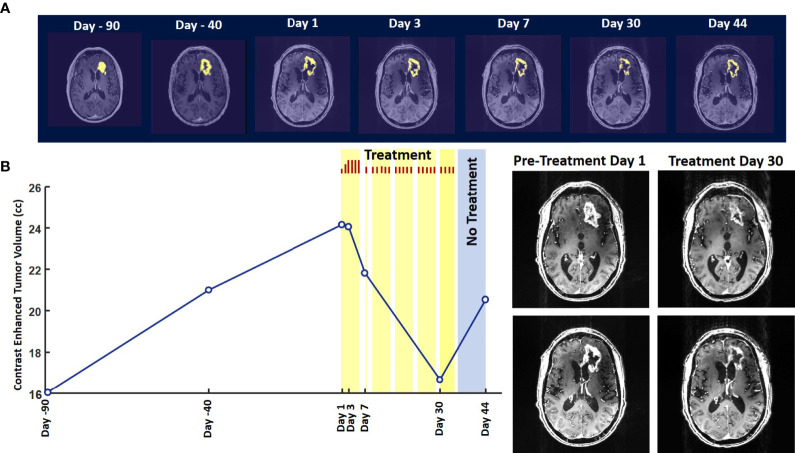
Change in Contrast-Enhanced Tumor Volume. **(A)** T1-weighted axial post-contrast scans showing the contrast-enhanced tumor (CET) highlighted with an overlayed automated computer program-generated light-yellow mask at different time points **(B)** Left **–** A graph showing the change in CET volume over time. The treatment times and durations are shown as red bars and light-yellow highlights. The long pause in treatment is shown as a light-blue highlight. Right – T1-weighted axial post-contrast scans showing CET at two levels along the dorso-ventral axis at Day 1 before treatment and Day 30 of treatment.

Evaluation of the T1 post-contrast clinical MRI scans obtained before initiation of treatment showed progression in accordance with the RANO criteria (**Figure 2A**). All scans acquired during treatment showed stable disease, according to these criteria (**Figure 2A**). To obtain an objective quantitative assessment of the CET volume we used an automated MATLAB software-based script. This analysis showed marked changes in CET volume with treatment. **Figure 2B** shows a plot of the CET volume as a function of time before and after initiation of treatment. It reveals that there was substantial growth of the tumor volume over the 3 months before the treatment. Within the first 3 days of treatment the trend is reversed with the volume steeply decreasing by ~10% on Day 7 and then less steeply by 31% on Day 30. Based on a Bayesian-type assessment of the probability of a decrease in CET volume at each post-treatment initiation time point, the decrease at Day 30 is statistically significant at P = 0.036. The treatment was paused on Day 37. After the pause we see another trend reversal and an increase in CET volume on Day 44.

**Figure 3:**
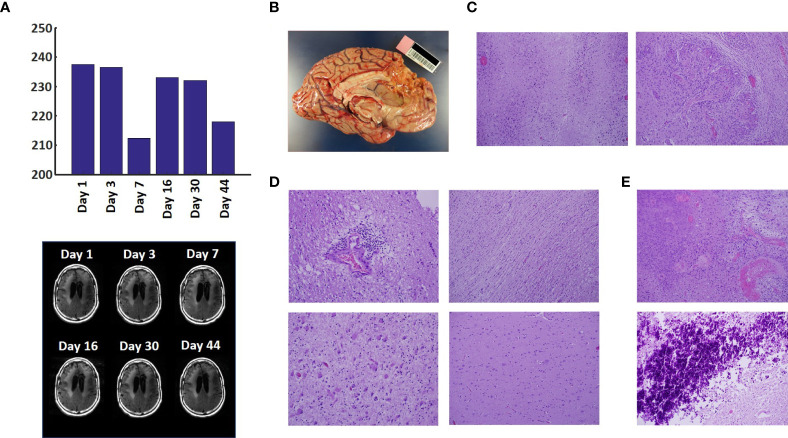
Variation in Enhanced Intensity Volumes in T2-FLAIR MRI Scans and Autopsy Findings. **(A)** Top – Bar plots of the volumes of T2-FLAIR intensity enhancement in the whole brain at different time points. Overall, there was up to 11% decrease in T2 FLAIR volume over the course of treatment. Bottom – Representative T2-FLAIR images are shown. **(B)** Left hemisphere of the brain, examined grossly, showing no tumor mass. **(C)** Photomicrographs of the left cortex showing bland necrosis, residual tumor, and microvascular proliferation with thick-walled vessels. **(D)** Top left – Microscopic field of the left cingulate cortex showing a focus of rarefied, perivascular inflammation. Bottom left – Cortical field showing rarefied parenchyma and residual tumor cells, enlarged with treatment-type effect that can be seen in GBM. Top right – Micrographic field of the corpus callosum showing thinned, rarefied white matter tract. Bottom right – Field showing relatively uninvolved contralateral (right) cortex. **(E)** Top – Micrographic field in the left cortex showing infarct-like necrosis (left), tumor (right), and fibrin thrombus (lower right). Bottom – Left cortical field showing necrotic tissue with dystrophic calcification.

The T2-FLAIR data in **Figure 3A** show changes in enhanced intensity volume of 1 – 11% over time. The decreases in volume are greater after a 3-day pause in treatment on Day 7 and after an 8-day pause on Day 44. These decreases are likely due to reduction in treatment-related cerebral edema and/or reduction in non-contrast enhancing tumor infiltration. The patient died ~3 months after cessation of treatment from the CHI. A brain only autopsy showed a resection cavity in the left frontal lobe (6.0 x 5.0 x 3.5 cm) and recurrent/residual glioblastoma with associated treatment effect (see **Figures 3B–E**). Residual/recurrent high-grade glioma was present, including foci of densely cellular tumor, focal microvascular proliferation, and necrosis (**Figure 3C**). In addition, there was prominent treatment effect with pallor and rarefaction of white matter (**Figure 3D**), reactive astrocytosis, infarct-like necrosis (**Figure 3E**) and bizarre nuclear atypia within residual tumor cells. Additional features of treatment effect included dystrophic calcifications (**Figure 3E**).

## Discussion

The findings of this study indicate that Oncomagnetic device-based OMF therapy is well tolerated by a patient who has end-stage recurrent GBM with leptomeningeal involvement and has no other available effective treatment options. They also demonstrate a clinically significant reduction in CET volume with reductions in non-enhanced tumor volume and/or edema in T2-FLAIR scans. The temporal profile of changes in CET volume also suggests a correlation with the treatment dose and the presence or absence of treatment. When the treatment dose was higher (6 hours/day for 4 days) we see a tumor volume reduction rate of 2.32 cm^3^/day. When it was lower (2 hours/day for 9 days and 3 hours/day for 18 days) the reduction is 1.03 cm^3^/day. Moreover, when the treatment was paused for 8 days the decreasing trend reversed and the CET volume increased, instead. Assuming that the ~1.03 cm^3^/day decreasing trend had continued until the treatment was paused, we can estimate that the CET volume grew at the rate of 1.26 cm^3^/day during the pause. Despite the apparent correlation it is possible that the treatment response is independent of the short-term changes in the treatment dose.

To our knowledge, there is no report in the literature of a noninvasive treatment-related shrinkage of CET volume of GBM at a rate comparable to that seen in this study. One published report on Optune™ therapy has reported that the time course of change in tumor volume in MRI scans shows a ~15% reduction over ~3 months (15). Besides Optune™, the other type of treatment approved by the FDA and recommended as a standard in National Comprehensive Cancer Network guidelines for recurrent GBM is the anti-vascular endothelial growth factor (VEGF) monoclonal antibody, Bevacizumab (16, 17). Bevacizumab treatment response of reduction in tumor volume on MRI scans has been reported to be lower than is observed in the present study (18). Furthermore, while anti-VEGF drugs in general have mild toxicity profiles and two Phase II trials have shown anti-tumor efficacy (19, 20), a subsequent Phase III trial did not show a significant increase in overall survival (21–23).

## Conclusion

Noninvasive Oncomagnetic device based OMF therapy appears to be a safe and efficacious new modality of treatment against GBM that potentially has many advantages over existing treatments. The present report has the limitation of the treatment being conducted in only a single patient so far. Extending it to more patients in research studies would provide additional information regarding safety and efficacy.

## Data Availability Statement

The original contributions presented in the study are included in the article/**Supplementary Material**. Further inquiries can be directed to the corresponding author.

## Ethics Statement

The studies involving human participants were reviewed and approved by Houston Methodist Research Institute Institutional Review Board. The patient/participant provided their written informed consent to participate in this study. Written informed consent was obtained from the individual for the publication of any potentially identifiable images or data included in this article.

## Author Contributions

SH and DB designed the study and drafted the manuscript. SH designed the device used in the study, supervised its construction and testing and quantitively analyzed the imaging data. DB provided medical care to the study subject, supervised the delivery of device treatment, and conducted his clinical assessments. SH, MS, and DB designed the device treatment protocol and interpreted the findings. LN constructed and tested the device and provided device treatment to the study subject. All authors contributed to the article and approved the submitted version.

## Funding

This work was supported by a grant from the Translational Research Initiative of the Houston Methodist Research Institute to SH and DB, and by Donna and Kenneth Peak, the Kenneth R. Peak Foundation, the John S. Dunn Foundation, the Taub Foundation, the Blanche Green Fund of the Pauline Sterne Wolff Memorial Foundation, the Kelly Kicking Cancer Foundation, the Gary and Marlee Swarz Foundation, the Methodist Hospital Foundation, and the Veralan Foundation. The John S. Dunn Foundation also supports the Distinguished Professorship of MS.

## Conflict of Interest

SH, MS, and DB are listed as inventors on a U.S. patent application filed by Houston Methodist Hospital for the device used in this report.

The remaining author declares that the research was conducted in the absence of any commercial or financial relationships that could be construed as a potential conflict of interest.
