# Supplementary material for: Case Report: End-Stage Recurrent Glioblastoma Treated With a New Noninvasive Non-Contact Oncomagnetic Device
Source: Front Oncol. 2021 Jul 22;11:708017. doi: 10.3389/fonc.2021.708017 (PMC8341943; doi:10.3389/fonc.2021.708017)
Supplement: Supplementary file 1 [file DataSheet_1.pdf]

Supplementary Appendix

Supplementary Methods

Oncomagnetic Device Details

The microprocessor in the device controller runs a software program that activates the oncoscillators sequentially for specified durations and with precise timings, to achieve an effective maximal frequency. The helmet is worn by the patient over a tight-fitting neoprene cap. The first oncoscillator was attached to the helmet at a left frontal location corresponding to the location of the tumor, the second at a symmetrical location on the contralateral side, and the third in the midsagittal plane at the parieto-occipital junction.

Data Processing, Normalization and Plotting

The DICOM images were converted to the NifTi 3D volume format using MRIcron (Chris Rorden, McCausland Center for Brain Imaging) software. Post-contrast scans at each post-treatment initiation time point were co-registered with the pre-treatment Day 1 scan using Statistical Parametric Mapping version 12 (SPM 12) software (Institute of Neurology, University College London). The co-registered scans were then imported into MATLAB (Mathworks, Natick, MA) and contrast-enhanced tumor (CET) volume estimated using an in-house MATLAB script and a uniform normalized intensity threshold. The script automatically counted all voxels that had contrast intensities above the same threshold in the same manually defined rectangular box containing the CET at each time point and multiplied the number of such voxels by the volume of each voxel in cm^3. The same automated procedure was followed for calculating the enhanced intensity region in the T2 FLAIR images. The mean volume was then plotted as a function of time. The pre- and post-treatment initiation T2-FLAIR scans were co-registered in the same manner as above with the pre-treatment T1 post-contrast scan. However, the co-registered scans were subjected to skull-stripping using the segmentation tool of SPM 12 and a MATLAB script. The volume of enhanced intensity in T2-FLAIR was then estimated by normalized intensity thresholding using the MATLAB script, and a bar plot of the enhanced intensity volume in the whole brain was plotted with respect to time.
